# Impact of percutaneous coronary intervention in right coronary artery on right ventricular function in patients with acute myocardial infarction

**DOI:** 10.21542/gcsp.2022.15

**Published:** 2022-12-30

**Authors:** Malladi Srinivasa Rao

**Affiliations:** 1 Department of Cardiology, Government General Hospital, Guntur, Andhra Pradesh, India

## Abstract

**Background:** Right ventricular (RV) dysfunction is a potent predictor of mortality and morbidity following acute myocardial infarction (AMI). Despite the fact that elective percutaneous coronary intervention (PCI) has significantly decreased myocardial damage to the left ventricle, there is a lack of information regarding the effect of PCI on RV function.

**Aim:** This study aimed to examine the effect of right coronary artery (RCA) revascularization on the systolic and diastolic functions of the right ventricle following acute inferior wall myocardial infarction.

**Methods:** Fifty-nine patients diagnosed with acute inferior wall myocardial infarction after RCA revascularization were prospectively investigated between April 2018 and January 2020. The patients underwent 2D echocardiography. RV systolic and diastolic functions were reported before and after the PCI procedure and compared using echocardiographic RV systolic and diastolic parameters.

**Results:** After PCI, echocardiographic RV systolic and diastolic functions significantly improved in the proximal and mid RCA in terms of TAPSE, RVFAC, and E/A**.** Significant improvement was found in the mid RCA in terms of S′ velocity (*p* = 0.008) and in the proximal RCA in terms of E/e′ (*p* = 0.021). Overall echocardiographic systolic and diastolic parameters in patients with RV dysfunction following PCI were improved [TAPSE (37.29% vs. 81.82%), S′ velocity (37.29% vs. 68.18%), RVFAC (33.90% vs. 90.00%), and E/A (33.90% vs. 75.00%)].

**Conclusion:** Our findings revealed that patients with RV dysfunction showed remarkable improvement after RCA revascularization. Hence, in future cases, RCA revascularization may become an appropriate treatment alternative for the recovery of patients with RV dysfunction.

## Introduction

Acute myocardial infarction (AMI) is a major cause of coronary heart disease despite breakthrough advancements in percutaneous coronary intervention (PCI) over the last few years^1^. Right ventricular (RV) dysfunction after AMI has emerged as a potent predictor for increased events of mortality and morbidity^2,3^. RV dysfunction has been reported in 50% of patients with inferior wall myocardial infarction and 10% of patients with anterior wall myocardial infarction^4,5^. Severe RV dysfunction is associated with right coronary artery (RCA) occlusion proximal to major RV branches^6,7^. Notably, RV dysfunction affects left ventricular function by limiting left ventricle preload. It also causes adverse effects on systolic and diastolic interactions through the intraventricular septum and pericardium (ventricular coupling)^8^. Therefore, the diagnosis of RV dysfunction is of utmost importance.

Although several studies have assessed the role of left ventricular function in the prognosis of patients with AMI, little attention has been paid to the role of systolic and diastolic RV functions. This can be due to diagnostic limitations of the electrocardiogram (ECG) and echocardiography of the irregularly shaped right ventricle^9,10^. To this end, this study aimed to examine the effect of RCA revascularization on systolic and diastolic functions of the right ventricle following acute inferior wall MI. It also analyzed the RV function response in each RCA segment following PCI.

## Methods

This single-center, prospective interventional study comprised 59 adult patients (age >18 years) who were referred to a tertiary healthcare center between April 2018 and January 2020. Patients who were diagnosed with acute inferior wall myocardial infarction with an angiographic diagnosis of isolated RCA lesions following PCI were included, irrespective of the thrombolysis status.

The exclusion criteria in the study were:

1. stable ischemic heart disease,
2. angiographic evidence of significant lesion in arterial territory other than RCA,
3. history of chronic obstructive pulmonary disease or any other chronic respiratory conditions,
4. history of coronary artery bypass grafting or valvular surgery or previous PCI,
5. history of renal or hepatic failure, or
6. history of cardiomyopathy.

Demographic data, lesion characteristics, and echocardiographic parameters were also recorded. The endpoint of the study was the improvement in RV dysfunction following PCI. Written informed consent was obtained from all the patients.

Prior to PCI, patients were evaluated by 2D echocardiography using the Esoate MyLab™X7 machine system. Primary PCI was performed using an ultrathin (60 µm) Supralimus Grace sirolimus-eluting stent (Sahajanand Medical Technologies, Surat, Gujarat) within 24 h of 2D echocardiography. After PCI, 2D echocardiography was performed with the Esoate MyLab™X7 machine system within 24-48 h. Left ventricular function was assessed as the percent change in ventricular cavity area from end-diastole to end-systole, and the left ventricular ejection fraction (LVEF) was evaluated^11^.

The following parameters were measured to assess RV function in the RV-focused apical four-chamber view:

1. tricuspid annular plan systolic excursion (TAPSE) was measured using M-mode echocardiography with the cursor optimally aligned along the direction of the lateral tricuspid annulus,
2. right ventricular fractional area change (RVFAC) was calculated using the following formula: (end-diastolic area × end-systolic area)/end diastolic area,
3. pulsed Doppler S wave (cm/s) and color tissue Doppler S wave (cm/s) measured peak systolic velocity (S′ velocity) of tricuspid annulus,
4. Doppler velocities of tricuspid E (early RV filling velocity) and A (late RV filling velocity) were evaluated,
5. tissue Doppler imaging study measured e’(early diastolic tricuspid annulus velocity) and a’(late diastolic tricuspid annulus velocity), thus evaluating the E/e′ ratio.

The reference limits of all echocardiographic parameters were adopted from the recommendations established by the joint venture of the American Society of Echocardiography and European Association of Cardiovascular Imaging. According to these guidelines, the abnormality threshold criteria for RV systolic dysfunction parameters were TAPSE < 17 mm, RVFAC < 35%, and S′ velocity < 10 cm/s. On the other hand, the abnormality threshold criteria for RV diastolic dysfunction parameters were E/A < 0.8 and E/e′ ≥ 4.0.11.

Quantitative data are presented as mean ± SD, and qualitative data are presented as frequencies. For categorical variables, comparisons between pre- and post-PCI were performed using McNemar’s test. Statistical significance was set at *p* value < 0.05. Statistical analyses were performed using SPSS statistical software version 15 (Statistical Package for the Social Sciences, Inc., Chicago, Illinois, USA).

## Results

Of the 59 patients, 41 (69.5%) were male and 18 (30.5%) were female. The mean age of the study population was 54 ± 8.2 years. No serious periprocedural complications were noted in any patient after successful PCI. Table 1 shows the baseline demographic and lesion characteristics of the study population. The mean LVEF of the study population was 58.6 ± 6.0%.

**Table 1:** Baseline demographic and lesion characteristics.

**Characteristics**	**Patients (*n* = 59)**
Age, (mean ± SD, years)	54 ± 8.2
Male, *n* (%)	41 (69.5%)
No. of lesions, n	59
Proximal RCA, *n* (%)	13 (22%)
Mid RCA, *n* (%)	34 (57.6%)
Distal RCA, *n* (%)	12 (20.3%)
Total occlusion, *n* (%)	9 (15.3%)
LVEF (%), mean ± SD	58.6 ± 6.0
Pre PCI e′/a′, mean ± SD	0.75 ± 0.14
Post PCI e′/a′, mean ± SD	0.81 ± 0.11

**Notes.**

RCA right coronary artery LVEF left ventricular ejection fraction PCI percutaneous coronary intervention SD standard deviation

Table 2 compares echocardiographic systolic parameters of the right ventricle before and after PCI. In all patients, RV function based on TAPSE improved statistically in the proximal (*p* = 0.031) and mid RCA (*p* = 0.004) following PCI. There was a significant difference in RV function after PCI in the mid RCA (*p* = 0.008) based on the S′ velocity. As per RVFAC, the improvement in RV function was found to be significant in the proximal (*p* = 0.004) and mid RCA (*p* = 0.016) following PCI.

**Table 2:** Comparisons of the systolic echocardiographic parameters of the right ventricle before and after PCI.

**Parameters**	**Proximal**		***p* value**	**Mid**		***p* value**	**Distal**		***p* value**
	**Pre (*n* = 13)**	**Post (*n* = 13)**		**Pre (*n* = 34)**	**Post (*n* = 34)**		**Pre (*n* = 12)**	**Post (*n* = 1)**	
**TAPSE**									
TAPSE < 17 mm RV dysfunction, *n* (%)	9 (69.2%)	3 (23.1%)	0.031	10 (29.4%)	1 (2.9%)	0.004	3 (25.0%)	0 (0.0%)	0.25
TAPSE ≥ 17 mm RV normal, *n* (%)	4 (30.8%)	10 (76.9%)		24 (70.6%)	33 (97.1%)		9 (75.0%)	12 (100%)	
**S′ velocity**									
S′ velocity ≤10 cm/s RV dysfunction, *n* (%)	9 (69.2%)	5 (38.5%)	0.219	10 (29.4%)	2 (5.9%)	0.008	3 (25.0%)	0 (0.0%)	0.25
S′ velocity >10 cm/s RV normal, *n* (%)	4 (30.8%)	6 (61.5%)		24 (70.6%)	32 (94.1%)		9 (75.0%)	12 (100%)	
**RVFAC**									
RVFAC ≥ 35% RV dysfunction, *n* (%)	9 (69.2%)	0 (0.0%)	0.004	9 (26.5%)	2 (5.9%)	0.016	2 (16.7%)	0 (0.0%)	0.5
RVFAC < 35% RV normal, *n* (%)	4 (30.8%)	13 (100%)		25 (73.5%)	32 (94.1%)		10 (83.3%)	12 (100%)	


**Notes.**

§ TAPSE tricuspid annular plan systolic excursion RV right ventricular S′ velocity peak systolic velocity RVFAC right ventricular fractional area change

Echocardiographic diastolic parameters of the right ventricle before and after PCI are shown in Table 3. RV function improved significantly based on the E/A ratio in the proximal (*p* = 0.039) and mid RCA (*p* = 0.016) following PCI, whereas RV function was significant only in the proximal RCA (*p* = 0.021) based on the E/e′ ratio after PCI.

**Table 3:** Comparisons of the diastolic echocardiographic parameters of the right ventricle before and after PCI.

**Parameters**	**Proximal**		**p value**	**Mid**		**p value**	**Distal**		**p value**
	**Pre (*n* = 13)**	**Post (*n* = 13)**		**Pre (*n* = 34)**	**Post (*n* = 34)**		**Pre (*n* = 12)**	**Post (*n* = 1)**	
**E/A**									
E/A < 0.8 RV dysfunction, *n* (%)	10 (76.9%)	3 (23.1%)	0.039	7 (20.6%)	0 (0.0%)	0.016	3 (25.0%)	2 (16.7%)	1.0
E/A ≥ 0.8 RV normal, *n* (%)	3 (23.1%)	10 (76.9%)		27 (79.4%)	34 (100%)		9 (75.0%)	10 (83.3%)	
**E/e’**									
E/e′≥ 0.4 RV dysfunction, *n* (%)	11 (84.6%)	3 (23.1%)	0.021	33 (97.1%)	31 (91.2%)	0.5	12 (100%)	10 (83.3%)	0.5
E/e′< 0.4 RV normal, *n* (%)	2 (15.4%)	10 (76.9%)		1 (2.9%)	3 (8.8%)		0 (0.0%)	2 (16.7%)	

**Notes.**

§ E/A early RV filling velocity / Late RV filling velocity E/e′ early RV filling velocity/ early diastolic tricuspid annulus velocity PCI percutaneous coronary intervention RV right ventricular

Table 4 shows the overall changes in echocardiographic systolic parameters in patients with RV dysfunction after PCI**.** RV function was improved in 81.82% of patients with TAPSE < 17 mm, in 68.18% of patients with S′ velocity ≤10 cm/s, and in 90.00% of patients with RVFAC < 35%. There was an overall improvement in RV function in echocardiographic diastolic parameters in 75.00% patients for E/A < 0.8 following PCI, whereas 14.29% patients showed unremarkable improvement for E/e′ ≥ 0.4, as demonstrated in Table 5.

**Table 4:** Changes in the echocardiographic systolic parameters in patients with RV dysfunction after PCI.

**Parameters**	**Pre PCI patients with RV dysfunction**	**Patients improved following PCI**
TAPSE < 17 mm	22/59 (37.29)	18/22 (81.82)
S′ velocity ≤10 cm/s	22/59 (37.29)	15/22 (68.18)
RVFAC < 35%	20/59 (33.90)	18/20 (90.00)

**Notes.**

§  TAPSE tricuspid annular plan systolic excursion PCI percutaneous coronary intervention RV right ventricular S′ velocity peak systolic velocity RVFAC right ventricular fractional area change

**Table 5:** Changes in the echocardiographic diastolic parameters in patients with RV dysfunction after PCI.

**Parameters**	**Pre PCI patients with RV dysfunction**	**Patients improved following PCI**
E/A < 0.8	20/59 (33.90)	15/20 (75.00)
E/e′≥ 0.4	56/59 (94.92)	8/56 (14.29)

**Notes.**

§ E/A early RV filling velocity / Late RV filling velocity E/e′ early RV filling velocity/ early diastolic tricuspid annulus velocity PCI percutaneous coronary intervention RV right ventricular

## Discussion

In the present study, systolic and diastolic echocardiographic parameters of RV function following RCA revascularization significantly improved. However, this improvement was observed in the proximal and mid parts of the RCA in TAPSE, RVFAC, and E/A. Significant improvement was found in the proximal part of the RCA for E/e′ and the mid part of the RCA for S′ velocity. Nonetheless, no improvement was observed in the distal part of the RCA for all parameters. Likewise, Nikdoust et al. reported a significant improvement in proximal RCA revascularization^12^. Furthermore, our study showed rapid recovery of RV function in AMI patients after PCI, which was also reported by Popescu et al. ^13^. This shows the importance of revascularization of the affected RCA.

The right ventricle is different from the left ventricle with respect to various anatomic and physiologic characteristics, yet they are related to each other. The shape, size, and conformity of one ventricle influences the hemodynamic features of the other ventricle, which is known as ‘ventricular coupling’^14^. Being a part of the right ventricle, the interventricular septum is influenced more by the left ventricle than the right ventricle, irrespective of advanced RV dysfunction or severe pulmonary hypertension. Moreover, involvement of the interventricular septum in evaluation of global RV function represents a great challenge^15^. Approximately 66% of the right ventricle is perfused with blood by the RCA in 85% of the world’s population through the posterolateral, atrioventricular nodal, and posterior descending arteries. The inferior wall of the right ventricle is easily affected by ischemia, whereas the infundibulum and anterior wall are unaffected segments^14^.

Owing to the fact that the severity of RV dysfunction is based upon the region of RCA occlusion, data pertaining to the lesion sites, collateral vessels, and dominance/ codominance is of paramount importance.

In a previous study, Hsu SY et al. al.. also stated that the region of coronary artery involvement might have a significant impact on RV function. Patients who were suffering from inferior infarction and without concomitant RV infarction, major echocardiographic parameters of RV function were identical in healthy individuals except for RV diastolic function. On the other hand, the variation in global RV function has been more prominent in patients with anterior wall infarction^16^.

Many previous studies have shown a significant association between echocardiographic parameters of RV function and LVEF^17–19^. Conversely, the present study did not investigate any correlations between echocardiographic parameters of RV function and LVEF. Furthermore, the recovery of RV function was significant following revascularization for all echocardiographic parameters. These parameters are independent predictors of RV function.

The present study has several limitations that need to be acknowledged. First, it was a single-center study with a small sample size; therefore, this study’s outcome may not be extrapolated to the general population. Second, patients with a single vessel (that is RCA) coronary artery disease were included in this study. Presumably, patients with multivessel coronary artery disease may adversely affect the RV function. Lastly, no investigation has been conducted on the size and length of the stents used during RCA revascularization.

## Conclusion

The findings of this study showed significant improvement in systolic and diastolic RV function after RCA revascularization in the proximal and mid RCA. Further studies with larger sample sizes and multivessel coronary artery disease are needed. In addition, the stent profile and anatomical features of the stenotic artery should be considered.
